# DNA methylation in bipolar disorder: mechanistic insights and translational challenges

**DOI:** 10.3389/fphar.2026.1871913

**Published:** 2026-07-24

**Authors:** Lu Sun, Ying Yang, Tong Liu, Hang-ran Zhu, Yi-ge Wang, Ya-fei Huang, Zi-xuan Lin, Yan-chen Feng, Fei-xiang Liu

**Affiliations:** 1 Encephalopathy Center, The First Affiliated Hospital of Henan University of Chinese Medicine, Zhengzhou, China; 2 The First Clinical Medical School, Henan University of Chinese Medicine, Zhengzhou, China; 3 The Third Clinical Medical School (College of Acupuncture and Tuina), Henan University of Traditional Chinese Medicine, Zhengzhou, China; 4 Joint Institute of Management and Science University at Henan University of Chinese Medicine, Zhengzhou, China; 5 Henan Provincial Engineering Research Center for Intelligent Applications in Traditional Chinese Medicine Internet Hospitals, Zhengzhou, China; 6 Henan University of Chinese Medicine Traditional Chinese Medicine (Zhongjing College), Zhengzhou, China; 7 Collaborative Innovation Center of Prevention and Treatment of Major Diseases by Chinese and Western Medicine, Zhengzhou, Henan, China

**Keywords:** bipolar disorder, DNA methylation, epigenetic regulation, neuroplasticity, neurotransmitter systems

## Abstract

Bipolar disorder (BD) is a severe and recurrent psychiatric disorder characterized by alternating manic, hypomanic, and depressive episodes, frequent comorbidity, high relapse rates, and an increased risk of suicide. Although the pathophysiology of BD remains incompletely understood, increasing evidence suggests that DNA methylation may represent an important epigenetic regulatory layer involved in BD-related biological heterogeneity. DNA methylation alterations have been reported in genes related to dopamine, serotonin, glutamate, and gamma-aminobutyric acid (GABA) systems, suggesting a potential role in neurotransmitter dysregulation. In addition, methylation changes in genes involved in neurotrophic signaling and ion-channel function may contribute to altered neuroplasticity and neuronal excitability. Clinically, candidate methylation signatures, including brain-derived neurotrophic factor (BDNF)-related methylation changes, specific GRIN2B CpG sites, and epigenetic age acceleration (EAA), have attracted attention for their potential relevance to diagnostic differentiation, disease progression, and treatment-response research. However, these signatures have not been clinically validated, and their reproducibility, tissue specificity, and longitudinal stability remain uncertain. Mood stabilizers, including lithium, valproate, and atypical antipsychotics, may partly influence methylation-related pathways, although their epigenetic effects and clinical significance remain incompletely defined. DNA methylation-targeted interventions remain experimental and require further validation regarding specificity, safety, blood–brain barrier delivery, and clinical applicability. This review summarizes current evidence on DNA methylation abnormalities in BD, focusing on neurotransmitter systems, neuroplasticity, ion-channel excitability, circadian rhythm, immune-inflammatory regulation, candidate methylation signatures and translational challenges. We emphasize that most available findings remain associative rather than causal, and future longitudinal, brain-region-specific, cell-type-resolved, and multi-omics studies are needed to clarify the mechanistic and translational relevance of DNA methylation in BD.

## 1 Introduction

BD is a complex and recurrent psychiatric disorder characterized by alternating episodes of mania or hypomania and depression (Oliva et al., 2024). Manic or hypomanic episodes are typically characterized by abnormally elevated or irritable mood, increased energy or activity, inflated self-esteem, decreased need for sleep, impulsivity, and risk-taking behavior, whereas depressive episodes commonly involve persistent low mood, anhedonia, reduced energy, impaired concentration, and, in severe cases, suicidal ideation or behavior (American Psychiatric Association, 2022; World Health Organization, 2019). BD usually emerges in late adolescence or early adulthood and is associated with frequent relapse, high psychiatric and medical comorbidity, and substantial functional impairment (Vieta et al., 2018). Epidemiological studies have estimated the lifetime prevalence of BD to be approximately 2.4%, with a lifetime suicide attempt rate of approximately 29.2% (Zhong et al., 2024; Dong et al., 2019). Therefore, BD imposes a considerable burden on patients, families, healthcare systems, and society (McIntyre et al., 2020; Gaine et al., 2019).

Current treatment strategies for BD primarily include pharmacotherapy, psychotherapy, and psychosocial interventions. Pharmacological treatment includes mood stabilizers, atypical antipsychotics, and, in selected cases, adjunctive antidepressants; however, treatment response varies substantially across individuals (Nierenberg et al., 2023). Studies on the efficacy of lithium treatment show that about one-third or more of patients do not respond well, and about half of patients require further treatment or experience relapse during long-term follow-up. Additionally, the efficacy criteria used in different studies are inconsistent (Ulrichsen et al., 2024). Other mood stabilizers, such as lamotrigine or valproate, and atypical antipsychotics may be effective in specific clinical phases, but their long-term effectiveness, tolerability, and safety profiles remain important clinical considerations (Simonetti et al., 2020). Overall, relapse prevention, treatment-response heterogeneity, adverse effects, and the lack of reliable predictors of therapeutic outcomes continue to represent major challenges in BD management. These limitations highlight the need to better understand the biological mechanisms underlying disease heterogeneity and treatment response.

DNA methylation is a key epigenetic modification in which a methyl group is added to cytosine residues, primarily at CpG sites, through the activity of DNA methyltransferases (DNMTs). Generally, promoter-region hypermethylation is often associated with transcriptional repression, whereas the functional consequences of low methylation or methylation changes in gene bodies and other regulatory regions are context-dependent (Moore et al., 2013). In the nervous system, DNA methylation has been implicated in neural development, synaptic plasticity, learning, memory, and activity-dependent gene regulation (Younesian et al., 2022; Peng et al., 2021). These methylation-related processes may regulate synapse formation and plasticity, thereby influencing signal transduction and neurotransmitter-system function. Such changes may be relevant to memory, behavioral regulation, and BD-related neurobiological abnormalities, although their direct relationship with clinical symptoms remains to be further clarified. More importantly, DNA methylation is reversible and modifiable, making it a potential regulatory target for mechanistic and therapeutic research. However, whether precise modulation of abnormal methylation can safely and effectively improve gene-expression dysregulation in BD remains to be established (Bundo et al., 2021).

**FIGURE 1:**
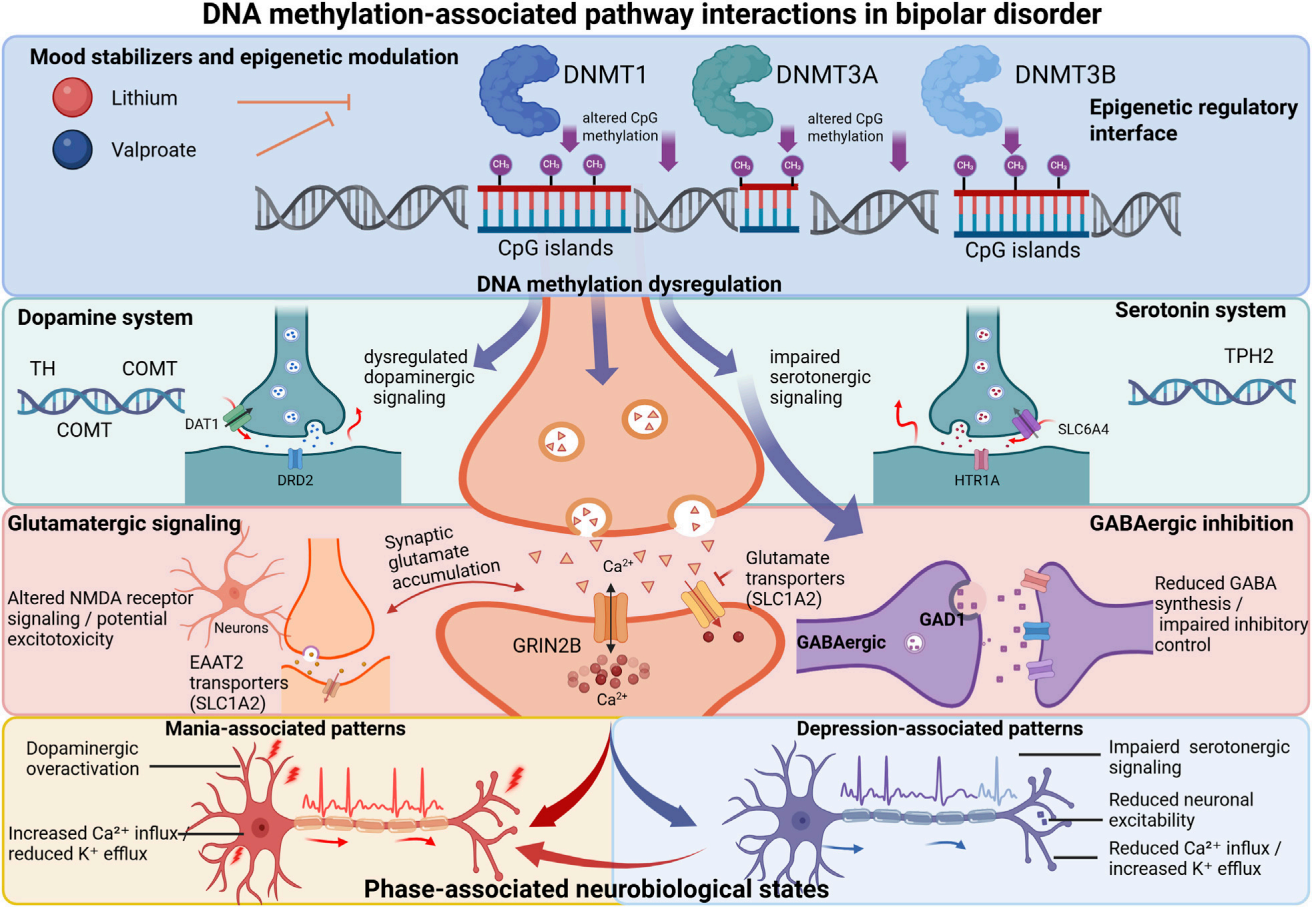
Conceptual model of DNA methylation-associated pathway interactions in bipolar disorder. This schematic illustrates a systems-level conceptual framework linking DNA methylation alterations with major biological pathways implicated in BD, including monoaminergic signaling, glutamate–GABA balance, neuroplasticity, ion-channel excitability, circadian rhythm regulation, and immune-inflammatory activation. DNA methylation is presented as a dynamic regulatory interface rather than a single upstream causal driver. Arrows indicate potential associations and pathway interactions rather than direct causality. This figure is intended to summarize pathway interconnectivity and does not imply that methylation changes directly cause specific clinical phenotypes. The lower panel distinguishes mania-associated and depression-associated neurobiological patterns to illustrate phase-related differences in neurotransmitter signaling and neuronal excitability; these labels represent conceptual associations rather than definitive phase-specific mechanisms. Created in BioRender. Sun et al (2026) https://www.biorender.com/w7y21uh.

**FIGURE 2:**
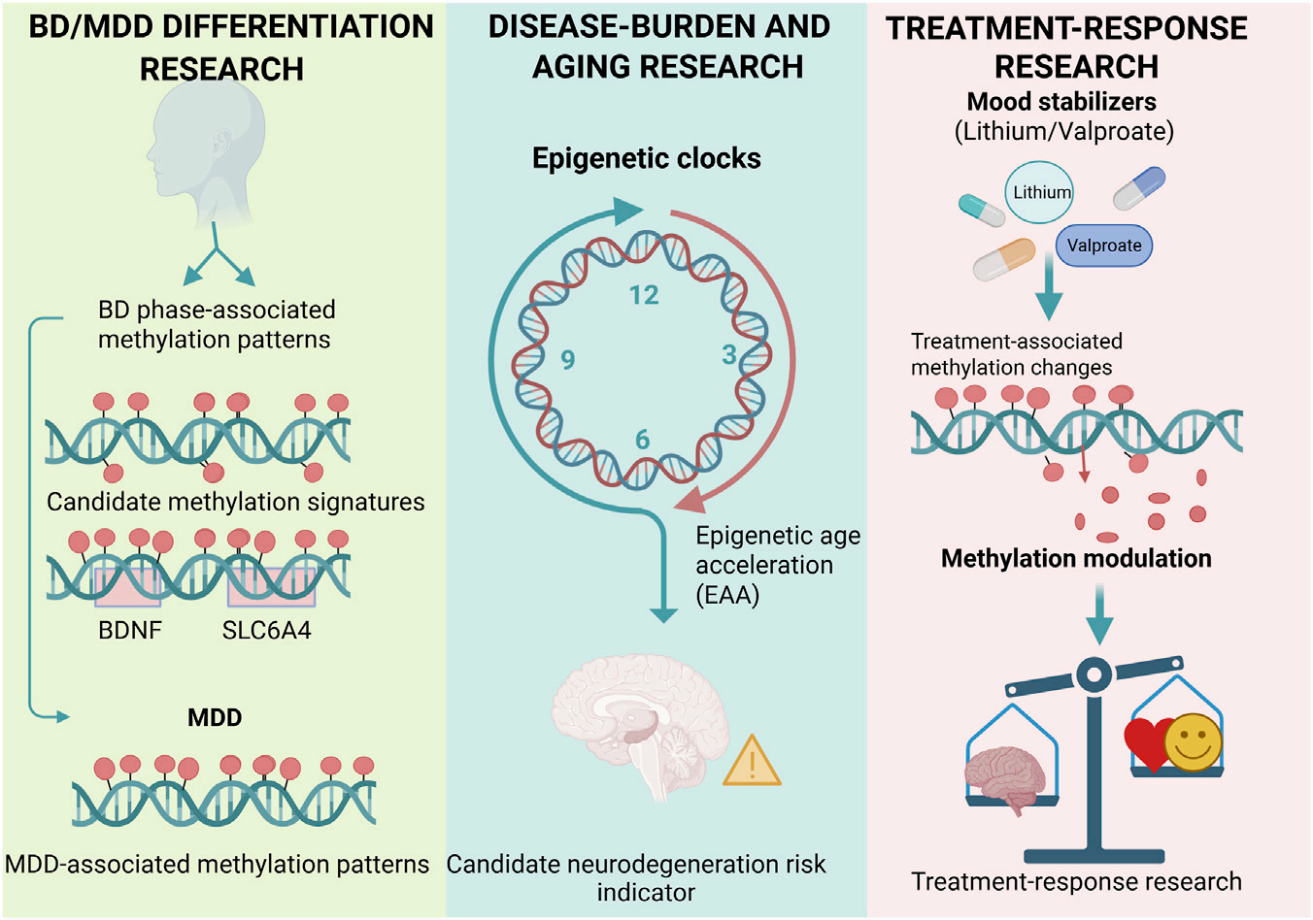
Exploratory translational relevance of candidate DNA methylation signatures in BD research. This schematic summarizes the potential translational relevance of DNA methylation research in BD, including candidate methylation signatures for BD/MDD differentiation research, epigenetic age acceleration (EAA)-related indicators for disease-burden and biological-aging studies, and treatment-associated methylation changes. These potential applications remain investigational and require independent validation before clinical implementation. The figure is intended to illustrate research directions rather than established diagnostic, prognostic, or therapeutic tools. Created in BioRender. Sun et al (2026) https://www.biorender.com/c81vhog.

Based on these considerations, this review aims to summarize current evidence on DNA methylation abnormalities in BD and to address the following questions: (1) How may DNA methylation alterations influence synaptic plasticity and neural network stability? (2) How might methylation-associated dysregulation of neurotransmitter systems contribute to emotional dysregulation and cognitive impairment in BD? (3) How should methylation findings from postmortem brain tissue and clinically accessible peripheral samples be interpreted differently when evaluating candidate signatures for BD stratification, treatment-response research, or relapse-risk assessment? By integrating evidence across neurotransmitter systems, neuroplasticity, ion-channel excitability, circadian rhythm, immune inflammation, and candidate translational applications, this review discusses the potential regulatory role of DNA methylation in BD pathophysiology and highlights key methodological challenges for future mechanism-informed biomarker and therapeutic research. The major DNA methylation alterations associated with bipolar disorder and their potential pathophysiological implications are summarized in Table 1. Candidate translational applications and current limitations are summarized in Table 2. The conceptual framework illustrating the relationship between DNA methylation, molecular mechanisms, and bipolar disorder pathophysiology is presented in Figures 1, 2.

**TABLE 1:** DNA methylation abnormalities of key genes in bipolar disorder and their potential pathophysiological or clinical associations.

System/Pathway	Key gene	Tissue/Sample type	Methylation status in BD	Reported/Potential effect on gene expression	Potential pathophysiological/Clinical associations
Dopamine system	COMT	Prefrontal cortex (PFC)	Low methylation at promoter	Increased expression	Altered dopamine metabolism; may be associated with cognitive and emotional dysregulation (Abdolmaleky et al., 2006)
	DAT1/SLC6A3	Prefrontal cortex (PFC), striatum, nucleus accumbens	Hypermethylation	Decreased expression	Reduced presynaptic dopamine reuptake and altered synaptic dopamine availability; may be associated with manic-state-related excitability and reward-circuit dysregulation (Salatino-Oliveira et al., 2018; Hasler et al., 2006)
Serotonin system	TPH2	Neurons in cortico-limbic structures	High methylation at promoter	Reduced mRNA expression	Reduced serotonin (5-HT) synthesis; may be associated with emotional instability, anxiety, and impulsivity (Fries et al., 2016; Zhang et al., 2015; Campos et al., 2011; Carrard et al., 2011)
Glutamate system	GRIN1	Dorsolateral prefrontal cortex (DLPFC)	High methylation at promoter	Suppressed expression	Altered NMDA receptor-related signaling; may be associated with cognitive impairment (Bundo et al., 2021)
	GRIN2B	Prefrontal cortex (PFC)	High methylation at promoter	Reduced expression	Altered synaptic transmission and NMDA receptor-related plasticity; may be associated with cognitive deficits, anxiety, and insomnia (Yu et al., 2025a)
GABA system	GAD1	Prefrontal cortex (PFC), hippocampus	High methylation at promoter	Reduced GAD67 expression	Reduced GABA synthesis; may contribute to excitatory–inhibitory imbalance (Ruzicka et al., 2015)
Neurotrophic factors	BDNF	Peripheral blood mononuclear cells (PBMCs)	High methylation at promoter/Val66Met-related sites	Reduced protein levels	Altered BDNF-related neuroplasticity; decreased serum BDNF levels have been associated with symptom severity (D’Addario et al., 2012)
Ion channels	CACNA1C	Peripheral blood	High methylation at intron	Reduced CaV1.2 expression	Altered calcium-channel regulation; may affect neurotransmitter release and synaptic plasticity (Smedler et al., 2019; Starnawska et al., 2016a)
Immune Inflammation	SOCS1	Peripheral blood	High methylation at promoter	Weakened anti-inflammatory function	Impaired negative regulation of the JAK/STAT pathway; may contribute to immune-inflammatory dysregulation (Aytac et al., 2023; Keshavarzi et al., 2019)
	HCG9	post‑mortem brain tissue	Low methylation	Increased expression	Altered pro-inflammatory cytokine regulation, including IL-6 and TNF-α; may be associated with immune-inflammatory activation (Pal et al., 2016)

**TABLE 2:** DNA methylation and exploratory translational relevance in bipolar disorder research.

Application area	Candidate methylation signature/Indicator	Sample/Tissue source	Methylation characteristics	Exploratory translational relevance	Evidence status and key limitations
BD/MDD differentiation research	BDNF and SLC6A4 methylation patterns	Peripheral blood/PBMCs; brain tissue evidence in selected studies	BD: phase-dependent methylation changes; MDD: relatively stable methylation patterns	Candidate signatures for BD/MDD differentiation research	Exploratory; affected by mood state, tissue source, medication exposure, and cohort heterogeneity (Fries et al., 2016; Ludwig and Dwivedi, 2016)
Disease stratification research	Genome-wide methylation profiles	Postmortem brain tissue and peripheral samples	BD: global hypomethylation with localized neuronal hypermethylation; MDD: broader hypermethylation tendency	Disease stratification and biological heterogeneity research	Not clinically validated for diagnosis or subtyping; tissue and cohort reproducibility remain uncertain (Bundo et al., 2021; Li et al., 2019)
Disease-burden and biological-aging research	Epigenetic age acceleration (EAA)	Peripheral blood-based epigenetic clocks; CSF-related indicators in selected studies	Accelerated epigenetic aging	Candidate indicator of illness burden and biological aging	Causality unclear; longitudinal validation required (Jeremian et al., 2022; Fries et al., 2026)
Treatment-response research	BDNF methylation levels	Peripheral blood/PBMCs and experimental models	High methylation; reduced BDNF-related signaling	Candidate marker for treatment-response studies	Not validated for efficacy prediction; independent longitudinal cohorts needed (Houtepen et al., 2016)
Drug-associated methylation research	SLC6A4 methylation levels	Patient-derived cellular models and peripheral blood	Drug-associated methylation modulation	Molecular readout of pharmacological exposure	Limited by *in vitro* evidence, peripheral samples, and medication confounding (Asai et al., 2013)
Experimental therapeutic research	DNA methylation-targeted interventions	Experimental, *in vitro*, and preclinical contexts	DNMT or methylation-state modulation	Future methylation-targeted therapeutic exploration	Experimental; limited by specificity, off-target effects, blood–brain barrier delivery, and safety concerns

A Systems-Level Mechanistic Framework Linking DNA Methylation to Bipolar Disorder Pathophysiology.

Rather than representing a single upstream cause of bipolar disorder (BD), DNA methylation may be better understood as a dynamic regulatory interface through which genetic vulnerability, environmental stress, inflammatory activation, circadian disruption, and pharmacological exposure converge to influence neural circuit function. In this framework, altered DNA methylation does not act in isolation. Instead, methylation changes in specific brain regions, cell types, and functional gene networks may interact with neurotransmitter signaling, synaptic plasticity, neuronal excitability, immune regulation, and circadian rhythm stability.

At the molecular level, dysregulated activity of DNA methyltransferases and TET-mediated demethylation machinery may contribute to region- and cell-type-specific methylation changes. These changes may affect genes involved in monoaminergic signaling, glutamate–GABA balance, neurotrophic support, and voltage-gated ion-channel function. At the circuit level, such molecular alterations may converge on prefrontal–limbic networks that are central to emotion regulation, reward processing, cognitive control, and sleep–wake stability. Therefore, dopamine, serotonin, glutamate, GABA, BDNF-related neuroplasticity, ion-channel excitability, circadian rhythm, and immune inflammation should not be interpreted as independent pathological modules, but as interconnected components of a broader epigenetic regulatory network.

Within this integrated model, neurotransmitter dysregulation may alter synaptic activity and calcium-dependent signaling, thereby influencing neuroplasticity-related pathways such as BDNF/TrkB and NMDA receptor signaling. Conversely, impaired neuroplasticity may further destabilize neurotransmitter systems and reduce the adaptive capacity of neural circuits. Circadian rhythm disruption may amplify mood-state instability by altering sleep–wake regulation, dopaminergic rhythmicity, and stress-response systems. Immune-inflammatory activation may also affect methylation regulation and synaptic function through cytokine-mediated effects on neurotrophic and glutamatergic pathways. These reciprocal interactions may help explain why BD is characterized by recurrent mood episodes, cognitive impairment, treatment heterogeneity, and phase-dependent biological changes.

Importantly, most currently available evidence remains associative rather than causal. Therefore, the mechanistic framework proposed in this review should be interpreted as a conceptual model that integrates existing findings and generates testable hypotheses, rather than as a definitive causal pathway. Future longitudinal, brain-region-specific, and single-cell methylome studies are required to determine whether these methylation changes precede disease onset, reflect mood-state transitions, or arise as consequences of illness progression, treatment exposure, or environmental factors.

## 2 BD and DNA methylation

DNA methylation is a dynamic epigenetic modification that involves the addition of a methyl group (CH_3_) to specific cytosine (C) nucleotides in the DNA molecule. This modification typically occurs at CpG sites, where cytosine is adjacent to guanine, and these sites are the primary targets of DNA methylation in the mammalian genome (Li et al., 2025; Jones, 2012). The methylation process is catalyzed by the DNA methyltransferase (DNMT) family. *DNMT1* is responsible for maintaining the established methylation state, while *DNMT3A* and *DNMT3B* mediate the establishment of new methylation. DNMT transfers the methyl group from the methyl donor S-adenosylmethionine to the 5′ carbon of cytosine, forming 5-methylcytosine. Accordingly, DNA demethylation can occur through two mechanisms: active and passive. Active demethylation is mediated by the Ten-Eleven-Translocation enzyme family, which oxidizes 5-methylcytosine (5-mC) to 5-hydroxymethylcytosine (5-hmC). These enzymes further oxidize 5-hmC to 5-formylcytosine and 5-carboxylcytosine, which are removed via base excision repair pathways. Passive demethylation occurs during cell division when *DNMT1* fails to replicate the methylation marks from the parental strand, leading to a gradual reduction in methylation levels in the daughter strand. DNA methylation is mainly concentrated in promoter regions, CpG islands, and gene body regions. Studies have shown (Suzuki and Bird, 2008; Brenet et al., 2011), methylation in promoter regions is often associated with gene expression repression, while methylation in gene bodies (exons and introns) is typically linked to transcriptional activity. Methylation in the first exon tends to be more associated with transcriptional silencing.

In individuals with BD, DNA methylation dysregulation is primarily manifested as an imbalance in DNMT expression and changes in methylation patterns. Research has found (Fries et al., 2016; Dong et al., 2015) in the prefrontal cortex of individuals with BD, there is increased binding of *DNMT1*, which is associated with increased methylation in the promoter regions of *GAD1*, *RELN*, and *BDNF* genes. Additionally, the *FAM63B* gene, which encodes a protein interacting with the kinesin-1 light chain involved in intracellular transport, shows significant hypomethylation in two CpG sites in BD. This suggests that DNMT abnormalities may contribute to its methylation disorder (Starnawska et al., 2016a). The altered methylation and expression of *FAM63B* may impair cellular transport function and contribute to the pathology of BD. Subsequent studies (Ho et al., 2019) have observed significant hypomethylation in gene promoters and other regulatory regions in brain areas such as the dorsolateral prefrontal cortex (DLPFC) and temporal pole (TP), suggesting that methylation dysregulation affects multiple genes and regulatory regions, rather than just individual genes. This brain region-specific pattern may relate to differences in cell type composition and function, with DLPFC hypomethylation linked to genes involved in emotion regulation, while *TP* hypomethylation is associated with genes related to language, speech, and social cognition. Additionally, there are also cell type-specific methylation differences between neurons and non-neuronal cells. Further analysis of promoter regions in individuals with BD shows generalized hypomethylation in both neuronal and non-neuronal cells. However, specific genes like *NTRK2* and *GRIN1* in neurons exhibit selective hypermethylation (Bundo et al., 2021).

In summary, DNA methylation abnormalities in BD appear to be multi-layered and context-dependent. Available evidence suggests that methylation dysregulation may occur at genome-wide, region-specific, and cell-type-specific levels, involving both broad promoter hypomethylation and selective hypermethylation of functionally important neuronal genes such as NTRK2 and GRIN1. These findings indicate that methylation alterations in BD should not be interpreted as uniform epigenetic changes across the brain or periphery. Rather, they may reflect a heterogeneous regulatory landscape shaped by brain region, cellular composition, mood state, environmental exposure, and treatment history.

From a systems-level perspective, such methylation changes may influence several interacting biological domains, including monoaminergic neurotransmission, glutamate–GABA balance, neurotrophic signaling, ion-channel excitability, circadian rhythm, and immune-inflammatory regulation. These domains are unlikely to act independently. Instead, they may converge on synaptic transmission, prefrontal–limbic circuit stability, and mood-state regulation. Accordingly, the following sections discuss DNA methylation-associated abnormalities not as isolated molecular events, but as interacting components of a broader regulatory network that may contribute to BD pathophysiology.

## 3 Neurotransmitter system dysregulation

### 3.1 DA system

DA plays multiple roles in the body, including motor control, reward and pleasure mechanisms, cognitive and emotional regulation, and endocrine control. The substantia nigra-striatum pathway controls motor function, the mesolimbic pathway mediates reward and pleasure, the mesocortical pathway regulates cognition and emotion, and the hypothalamic-pituitary pathway influences endocrine functions. Studies have shown (Bundo et al., 2021) that in the prefrontal cortex of individuals with BD, both neuronal and non-neuronal cells exhibit general hypomethylation in promoter regions, along with hypermethylation at specific loci. These findings suggest that epigenetic changes are unstable and brain-region specific.

An increasing number of studies indicate that dysfunction in the DA system may contribute to the pathophysiology of BD. Early studies by Abdolmaleky et al., 2006 reported that in the prefrontal cortex of individuals with BD, the promoter region of catechol-O-methyltransferase (*COMT*), a gene involved in DA metabolism, exhibits hypomethylation. This hypomethylation has been associated with increased *COMT* expression, potentially influencing DA metabolism and contributing to dysregulation of DA signaling pathways, which may lead to abnormal DA levels. This finding was subsequently supported by Nohesara et al. (2011). Accelerated DA metabolism and reduced DA concentration have been suggested to contribute to symptoms such as emotional numbness, loss of interest, lack of motivation, decision-making difficulties, attention deficits, and memory decline, as implicated by the DA hypothesis in BD (Ashok et al., 2017). In addition to metabolism-related genes, DA receptors are also regulated by methylation. The D2 receptor, encoded by the *DRD2* gene, mediates signal transduction through binding with DA. Studies have shown (Piao et al., 2022) increased methylation in the DRD2 promoter region is associated with alterations in the expression of genes involved in DA signaling, such as *COMT*, Dopamine Transporter 1 (*DAT1*), and *TH*. These changes may contribute to dysregulation of DA transmission. Additionally, alterations in the density or sensitivity of D2 receptors can further modulate DA signaling, potentially enhancing neuronal excitability. These changes are thought to contribute to the manifestation of mania-related symptoms, such as elevated energy, mood fluctuations, impulsivity, and, in severe cases, hallucinations.

At the level of DA transport, DAT1 methylation may be associated with symptom phases. DAT1 expression and DA-system function may be epigenetically regulated. Salatino-Oliveira et al. (2018) reported that individuals with BD exhibit differences in DAT1 expression and DA transport function compared with healthy controls, and these changes may be related to dysfunction in DA reuptake mechanisms. Reduced DAT1 expression may limit presynaptic DA reuptake, thereby potentially increasing DA availability in the synaptic cleft and affecting signal transmission. Elevated DA signaling may be associated with increased activation of reward-related and prefrontal cognitive circuits, a neurodynamic alteration that is consistent with excessive euphoria, hyperactivity, and impulsivity observed during manic episodes of BD (Hasler et al., 2006).

Methylation may also be involved in the rate-limiting step of DA synthesis. Methylation analysis of brain tissue from individuals with BD has shown that the methylation status of the tyrosine hydroxylase (TH) gene is significantly correlated with altered DA levels in the brain. In addition, hypomethylation in the enhancer region of the IGF2 gene has been proposed to enhance transcriptional regulation of TH, potentially influencing DA synthesis, which may be associated with manic-state-related excitement and impulsivity (Pai et al., 2019).

Taken together, methylation alterations in genes related to dopamine synthesis, metabolism, receptor signaling, and reuptake may contribute to dopaminergic dysregulation in BD. COMT promoter hypomethylation has been associated with altered dopamine metabolism, whereas methylation changes in DRD2, DAT1/SLC6A3, and TH-related regulatory regions may affect dopamine signaling, transport, and synthesis. These findings provide a plausible molecular framework for understanding mood-state-related alterations in reward processing, motivation, impulsivity, and cognitive control.

However, the available evidence should be interpreted cautiously. Most studies linking dopaminergic gene methylation to BD phenotypes are associative, and direct causal relationships among methylation changes, gene expression, dopamine availability, and clinical symptoms have not been fully established. Differences in tissue source, brain region, sample size, mood state, medication exposure, and environmental factors may contribute to inconsistent findings across studies. Therefore, methylation changes in dopamine-related genes should be considered candidate mechanisms rather than established drivers of manic or depressive symptoms.

### 3.2 Serotonin (5-HT) system

5-HT is a vital neurotransmitter in the brain, widely distributed across the cerebral cortex, limbic system, hypothalamus, and spinal cord. In the cerebral cortex, serotonin primarily regulates mood, cognition, and perception. In the limbic system, it is involved in emotional and memory regulation. In the hypothalamus, it controls appetite, body temperature, and endocrine functions, while in the spinal cord, it participates in pain and sensation regulation.

The synthesis of 5-HT begins with tryptophan, which is catalyzed by tryptophan hydroxylase (TPH) to produce 5-hydroxytryptophan (5-HTP), then converted to 5-HT (Kanova and Kohout, 2021). TPH2, specifically expressed in the central nervous system, is a rate-limiting enzyme and a key target for 5-HT synthesis. Studies indicate that TPH2 gene polymorphisms are associated with BD risk and may influence mood fluctuations and other clinical phenotypes by modulating 5-HT levels (Gao et al., 2016). TPH2 expression is subject to epigenetic regulation; elevated promoter methylation levels have been correlated with decreased mRNA expression. This downregulation potentially leads to reduced enzyme protein levels and lower 5-HT availability, which may contribute to emotional instability, anxiety, and increased impulsivity (Fries et al., 2016; Zhang et al., 2015; Campos et al., 2011; Carrard et al., 2011).

Additionally, both the serotonin transporter gene *SLC6A4* (encoding SERT) and the serotonin receptor gene *HTR1A* have been implicated in epigenetic dysregulation in BD. Elevated methylation of the *SLC6A4* promoter is associated with reduced expression of the transporter, which may weaken serotonin (5-HT) reuptake in the synaptic cleft, potentially affecting serotonergic signaling. These epigenetic modifications in the *HTR1A* and *SLC6A4* genes are believed to contribute to mood dysregulation and anxiety, core symptoms observed in BD, though further studies are needed to clarify their exact role in the pathophysiology of the disorder (Sugawara et al., 2011).

In summary, methylation alterations in serotonin-related genes may contribute to serotonergic dysregulation in BD by affecting 5-HT synthesis, receptor signaling, and transporter function. Elevated *TPH2* methylation has been associated with reduced 5-HT-related expression, whereas methylation changes in *HTR1A* and *SLC6A4* may influence receptor and transporter regulation. These alterations may provide candidate mechanisms linking serotonergic dysfunction with mood instability, anxiety, and impulsivity in BD. However, the available evidence remains largely associative, and the functional consequences of these methylation changes require further validation in brain-region-specific and longitudinal studies.

### 3.3 Glutamate system

The glutamate system plays a crucial role in the pathophysiology of BD, particularly in signal transduction mediated by N-methyl-D-aspartate (NMDA) receptors. *GRIN1* is a key gene encoding the NMDA receptor NR1 subunit, and its expression directly affects glutamate signaling efficiency. In individuals with BD, differential methylation in the *GRIN1* promoter region has been observed, which may affect transcription factor binding and reduce *GRIN1* transcription and NR1 synthesis. This could potentially weaken NMDA receptor-mediated glutamate signaling, which has been associated with cognitive impairment (Bundo et al., 2021). *GRIN2B* encodes the NMDA receptor NR2B subunit, which is crucial for synaptic plasticity and cognitive function. Several studies have shown (Yu et al., 2025a; Yu et al., 2025b) that individuals with BD exhibit high methylation levels in the *GRIN2B* promoter region. This methylation is not only associated with cognitive deficits during depressive episodes but also with anxiety and insomnia symptoms, suggesting that impaired NR2B function may play a significant role in BD’s neurobiological mechanisms. Additionally, the *SLC1A2* gene encodes the glutamate transporter excitatory amino acid transporter 2 (EAAT2), which is responsible for clearing glutamate from the synaptic cleft to maintain synaptic transmission balance. Jia et al. (2017) reported that individuals with BD exhibit elevated methylation in the SLC1A2 promoter, which may be associated with a reduction in EAAT2 expression. This limitation in presynaptic glutamate reuptake potentially leads to glutamate accumulation in the synaptic cleft. Such accumulation may enhance NMDA receptor activation, triggering excitotoxicity and disrupting neural circuit function. This sustained activation could not only disturb synaptic transmission balance but may also contribute to cellular damage and neural network dysfunction.

Taken together, methylation alterations in GRIN1, GRIN2B, and SLC1A2 may be involved in glutamatergic dysregulation and NMDA receptor-related dysfunction in BD. Altered methylation of GRIN1 and GRIN2B may affect NMDA receptor subunit expression and synaptic plasticity, whereas SLC1A2 promoter methylation may influence glutamate clearance through EAAT2 regulation. These findings provide a plausible epigenetic framework linking glutamatergic signaling abnormalities with cognitive impairment, affective instability, and sleep–wake disturbances in BD.

However, the interpretation of these findings requires caution. Current evidence does not establish a direct causal pathway from methylation changes in glutamate-related genes to NMDA receptor dysfunction or clinical symptoms. Reported associations vary across tissue sources, brain regions, CpG sites, cohort characteristics, mood states, medication exposure, and analytical methods. Moreover, peripheral methylation findings may not fully reflect glutamatergic regulation in disease-relevant brain circuits. Therefore, GRIN1, GRIN2B, and SLC1A2 methylation abnormalities should be considered candidate mechanisms that require further validation in longitudinal, brain-region-specific, and cell-type-resolved studies.

### 3.4 GABA system

GABA is widely distributed in the brain and spinal cord, playing a particularly important role in the cerebral cortex and hippocampus (Whissell et al., 2015). GABA regulates neuronal excitability in the cerebral cortex, collaborates with glutamate in the hippocampus to facilitate learning and memory, controls motor coordination and balance in the cerebellum, influences motor selection in the basal ganglia, and modulates sensory transmission and motor control in the spinal cord (Hori and Hoshino, 2012). In the GABA system, *GAD1* is a core gene involved in synthesis regulation, encoding the key enzyme *GAD67* involved in GABA synthesis. Studies show (Ruzicka et al., 2015) that individuals with BD exhibit specific hypermethylation of the *GAD1* promoter in the prefrontal cortex and hippocampus, which is associated with the suppression of *GAD1* transcription and reduced *GAD67* expression. This may contribute to impaired GABA synthesis, as the GABAergic system plays a key role in modulating neuronal activity and maintaining cognitive and emotional balance. Additionally, genes related to *GAD1* regulation, involved in chromatin regulation and cell cycle control, also exhibit methylation changes that may indirectly affect *GAD1* expression and function, further disrupting GABA synthesis (Huang and Akbarian, 2007).

Furthermore, multiple genes associated with GABA function show abnormalities in BD. In the hippocampus of individuals with BD, genes such as *MSX1*, *CCND2*, and *DAXX* exhibit significant methylation changes (Ruzicka et al., 2015). *MSX1* hypermethylation weakens its transcriptional activation of downstream *GAD1*, indirectly reducing GABA synthesis. *CCND2* hypomethylation may be associated with increased expression, accelerating the cell cycle of GABAergic neurons and affecting their proliferation and survival. *DAXX* hypermethylation may impair its role in ubiquitination/protein homeostasis regulation, affecting the turnover and membrane localization of GABA receptors, thus reducing inhibitory signal transmission efficiency. Together, these methylation changes may contribute to GABAergic dysfunction in BD, although their direct functional consequences remain to be further validated.

Altered methylation of GABA-related genes may add an inhibitory component to the broader excitatory–inhibitory imbalance implicated in BD. Rather than acting as an isolated pathway, GABA-related methylation abnormalities may interact with glutamatergic, dopaminergic, and serotonergic alterations to affect cortical and hippocampal circuit stability. This integrated view suggests that GABA-related methylation changes are best interpreted as candidate contributors to neurotransmitter-network dysregulation in BD, particularly through disruption of glutamate–GABA balance, rather than as independent causal mechanisms.

These neurotransmitter systems are unlikely to operate independently in BD. Dopaminergic and serotonergic dysregulation may influence reward processing, emotional reactivity, and stress responsiveness, whereas glutamate-GABA imbalance may affect excitatory-inhibitory stability and synaptic plasticity. These neurotransmitter abnormalities may converge on calcium-dependent signaling, NMDA receptor activity, and BDNF/TrkB-mediated neuroplasticity, thereby linking methylation-associated neurotransmitter changes with broader circuit-level instability.

## 4 Abnormal neuroplasticity and neurotrophins

Neuroplasticity refers to the brain and nervous system’s capacity for reversible structural and functional changes in response to injury, learning, or environmental adaptation. Neurotrophic factors are a class of secreted proteins that support neuron survival, growth, connectivity, and function. They belong to the neurotrophin family, including *BDNF*, Nerve Growth Factor (NGF), and Neurotrophin-3 (NT-3). These factors play a critical role in neuroplasticity, promoting neuron survival, protecting against degenerative changes, and supporting regeneration after injury.

### 4.1 BDNF methylation abnormalities

*BDNF* is a core member of the neurotrophin family and plays an essential role in neuroplasticity and emotional regulation (Lu, 2003). *BDNF* also participates in the formation and maintenance of neurotransmitter systems such as DA, 5-HT, GABA, and acetylcholine. It promotes presynaptic neurotransmitter release and enhances postsynaptic receptor sensitivity. These mechanisms improve synaptic signal transmission efficiency, influencing emotion regulation, cognitive abilities, and stress responses. Such processes are closely related to the pathogenesis of BD, as indicated by the association of *BDNF*/*BDNF-AS* gene variants with BD and other psychiatric disorders (Shkundin and Halaris, 2023).

In major depressive disorder, methylation changes in CpG sites upstream of *BDNF* exons have been investigated as candidate biomarkers related to disease progression and treatment response. Individuals with BD have also been reported to exhibit altered methylation in the *BDNF* promoter region. D’Addario et al. (2012) reported higher BDNF promoter methylation levels in individuals with BD than in healthy controls. Increased BDNF promoter methylation may be associated with reduced BDNF expression, which could contribute to impaired neuroplasticity, emotional dysregulation, and cognitive dysfunction. Clinical studies have also shown that serum BDNF levels may be lower during manic or depressive episodes and may correlate negatively with symptom severity (Munkholm et al., 2016). In animal models, deletion of BDNF promoter IV has been associated with altered expression of dopamine-, serotonin-, and norepinephrine-related genes in the frontal cortex and hippocampus, suggesting that BDNF deficiency may influence neurotransmitter-system regulation and mood-related behaviors (Sakata and Duke, 2014). In early-onset BD, increased methylation at BDNF Val66Met-related CpG sites has also been reported, suggesting a potential association between BDNF epigenetic regulation and disease susceptibility (Nassan et al., 2020). Furthermore, individuals with BD show specific changes in DNA methylation, including alterations in the *NTRK2* promoter, which encodes the TrkB receptor. This modification is associated with a reduction in TrkB expression, which may weaken the *BDNF* signaling pathway (Bundo et al., 2021).

Overall, methylation alterations involving the BDNF promoter, Val66Met-related sites, and the NTRK2 promoter may affect BDNF/TrkB signaling, neuroplasticity, and neurotransmitter-system regulation in BD. However, their direct functional consequences and clinical significance remain to be further validated.

### 4.2 Methylation abnormalities of NMDA receptor-related genes

NMDA receptors (NMDARs) are key ionotropic glutamate receptors (iGluRs) widely distributed in the central nervous system, mediating excitatory signal transmission between neurons. Their function is not limited to mediating rapid excitatory neurotransmission; they also help adjust the connections between neural networks, enabling the brain to continuously learn and adapt. NMDARs play a crucial role in learning, memory, and synaptic plasticity. Studies have shown (Clinton and Meador-Woodruff, 2004) individuals with BD often exhibit emotional fluctuations, cognitive impairments, and other symptoms, which are likely associated with abnormalities observed in NMDA receptors and related intracellular molecules.

The functional expression of NMDARs relies on their essential core subunit, GluN1, encoded by the GRIN1 gene. In individuals with BD, increased methylation in the GRIN1 promoter region has been observed in the prefrontal cortex, which may reduce GRIN1 expression and GluN1 subunit synthesis, potentially affecting NMDA receptor-related function. Altered NMDAR-related signaling may affect receptor composition, calcium influx, synaptic plasticity, and neuronal signal transmission efficiency. Such alterations may be associated with cognitive dysfunctions, including working memory deficits, impaired decision-making, decreased attention, and mood-state instability in BD (Bundo et al., 2021).

GRIN2B encodes the GluN2B subunit of the NMDAR, which is important for synaptic function and cognitive processes. Recent studies have reported abnormal methylation in the GRIN2B promoter region in individuals with BD (Yu et al., 2025a). Elevated GRIN2B methylation may reduce GRIN2B expression and contribute to NMDA receptor-related signaling abnormalities, which may be associated with cognitive impairment in BD. The GluN2B subunit is important for synaptic signal transmission, neuronal plasticity, and excitability. GRIN2B promoter hypermethylation may suppress GRIN2B transcription, reduce GluN2B protein expression, and potentially weaken NMDAR-related signaling. This may affect synaptic signal transmission and may be associated with impairments in learning, memory, and attention.

In addition, studies of individuals with BD and comorbid anxiety symptoms have reported abnormal methylation at specific CpG sites in the GRIN2B promoter region (Yu et al., 2024). For example, hypermethylation at CpG8 has been associated with poorer concentration, hypomethylation at CpG6 has been associated with poorer executive function, and hypermethylation at CpG9 has been associated with impaired verbal memory. These methylation abnormalities have been associated with cognitive dysfunction and may also be related to anxiety symptoms. These findings provide associative clinical evidence for a potential role of GRIN2B methylation abnormalities in cognitive and emotional regulation in BD.

In this context, methylation abnormalities in NMDA receptor-related genes may represent a potential cross-talk point between glutamatergic signaling and BDNF/TrkB-mediated neuroplasticity. Altered methylation of GRIN1 and GRIN2B may influence NMDA receptor-related signaling, which is closely linked to calcium-dependent pathways, CREB activation, and activity-dependent BDNF expression (Yu et al., 2025a; Lin et al., 1998). Through this potential mechanism, NMDA receptor-related methylation changes may affect synaptic plasticity, neuronal connectivity, and cognitive or emotional regulation in BD. Nevertheless, direct evidence demonstrating that these methylation changes drive progressive cognitive dysfunction or emotional dysregulation remains limited. Therefore, NMDA receptor-related methylation abnormalities should be interpreted as candidate mechanisms connecting glutamatergic dysfunction with impaired neuroplasticity rather than as established causal factors.

## 5 Ion channel abnormalities

The methylation status of key genes in voltage-gated ion channels can influence the transcription and expression of channel proteins. Abnormal ion channel function can indirectly regulate the activity and expression pathways of proteins involved in presynaptic neurotransmitter release. This may lead to an imbalance in synaptic transmission efficiency in BD, which contributes to emotional and cognitive dysfunction.

### 5.1 Calcium ion channels

Abnormalities in calcium ion-channel function may affect neurotransmitter release and synaptic plasticity. During manic episodes, increased Ca^2+^ influx may be associated with excessive neurotransmitter release, whereas reduced Ca^2+^ influx during depressive episodes may be related to insufficient neurotransmitter release. *CACNA1C* and *CACNB2* are key genes involved in L-type calcium-channel regulation, neuronal plasticity, cognitive function, and BD susceptibility.

*CACNA1C* encodes the α1C subunit (CaV1.2) of the L-type calcium channel, which is involved in Ca^2+^ transmembrane flux. Dysfunction of this channel may affect neuronal excitability, synaptic transmission, plasticity, learning and memory, and may also be related to neuronal differentiation, migration, and synapse formation. In BD, *CACNA1C* rs1006737 has been associated with changes in the thickness of the frontal and parietal cortical regions, suggesting that this variation may impact brain structure and function (Smedler et al., 2019; Rodríguez-Ramírez et al., 2020). Other studies have reported that individuals with BD carrying the rs1006737 risk allele may show increased cortical thickness after treatment with lithium or valproate, particularly in the right prefrontal cortex. This suggests a possible association with treatment-related neurobiological changes. Further studies have shown (Smedler et al., 2021), different *CACNA1C* risk alleles (such as rs2370411, rs1006737) are associated with peripheral BDNF levels. Animal studies suggest that activation of BDNF receptors may partially restore cognitive and synaptic abnormalities in *CACNA1C* heterozygous mice, supporting the potential mechanistic relevance of *CACNA1C* in neurodevelopment and synaptic function (Tigaret et al., 2021). At the epigenetic level, individuals with BD have been reported to exhibit hypermethylation in the third intron region of *CACNA1C,* suggesting altered methylation regulation at this locus. *CACNA1C* hypermethylation may affect transcription-factor binding and reduce transcriptional efficiency, potentially decreasing CaV1.2 expression and Ca^2+^ current. These changes may influence Ca^2+^-dependent neurotransmitter release and synaptic plasticity and may be associated with frontal-parietal cortical alterations and other brain-structure differences (Smedler et al., 2019; Starnawska et al., 2016b). However, direct links among CACNA1C methylation, channel dysfunction, electrophysiological changes, and BD pathophysiology require further validation.

*CACNB2* encodes the β subunit (CaVβ) of the L-type calcium channel, which regulates channel voltage dependence, activation/inactivation properties, and contributes to complex stability and localization. Reduced *CACNB2* expression may be associated with decreased Ca^2+^ influx, altered excitability, and impaired plasticity, and may affect the structure and function of the nervous system. Studies have found (Liu et al., 2019; Chen et al., 2020) individuals with BD carrying certain alleles of *CACNB2* show lower cortical thickness in the right prefrontal cortex compared to healthy controls and non-risk allele carriers, suggesting that this gene affects early changes in prefrontal structure. Other research shows (Jiang et al., 2023) these allele carriers exhibit increased resting-state functional connectivity (rs-FC) in regions such as the hippocampus and prefrontal cortex, suggesting a possible association with network dysfunction. At the epigenetic level, significant DNA methylation differences exist in the promoter and intron regions of *CACNB2* in specific haplotype carriers. These methylation changes may affect the activity of transcription factor binding sites. These findings suggest that these methylation differences may regulate the expression of *CACNB2* isoforms, thereby potentially contributing to BD-related neurobiological alterations (Walker et al., 2016).

### 5.2 Potassium ion channels

Potassium channel abnormalities may affect action potential repolarization and excitability homeostasis. During manic episodes, insufficient K^+^ efflux may be associated with prolonged neuronal excitation, whereas excessive K^+^ efflux during depressive episodes may be related to increased neuronal inhibition (Kaminsky et al., 2015). *KCNQ2* and *KCNQ3* regulate M-current (I_M), which is important for resting membrane potential and repolarization; their dysfunction may contribute to delayed repolarization and excitability imbalance, which may be relevant to BD-related neuronal instability.

In the prefrontal cortex of individuals with BD, reduced DNA methylation in the *KCNQ3* exon 11 region has been associated with decreased *KCNQ3* mRNA expression. Hypomethylation may reflect epigenetic regulation changes at this site, which could affect the transcription and splicing of the *KCNQ3* gene (Kaminsky et al., 2015). Newly identified *KCNQ2* splice variants may disrupt interactions with protein phosphatase 2 A subunit Bγ, potentially reducing channel stability and increasing neuronal excitability. This dominant-negative effect of *KCNQ2* splice variants may be related to excitability imbalance observed during manic episodes. Overall, methylation- or splicing-related changes in *KCNQ2* and *KCNQ3* may be associated with altered potassium-channel function, thereby potentially affecting neuronal excitability and repolarization.

Methylation-associated alterations in calcium- and potassium-channel-related genes may provide a candidate epigenetic mechanism for neuronal excitability dysregulation in BD. Changes in CACNA1C and CACNB2 methylation may influence L-type calcium-channel expression or function, thereby affecting calcium-dependent neurotransmitter release, synaptic plasticity, and activity-dependent gene transcription. Similarly, methylation or splicing-related changes in KCNQ2 and KCNQ3 may affect potassium-channel stability, repolarization, and excitability homeostasis. These alterations may converge on neuronal firing patterns and synaptic transmission efficiency, which are relevant to mood-state instability and cognitive dysfunction.

However, the proposed phase-related excitability model remains largely hypothetical. Although calcium- and potassium-channel abnormalities are biologically plausible contributors to manic and depressive states, direct evidence linking DNA methylation changes to channel protein expression, electrophysiological alterations, and specific clinical phases in BD remains limited. Existing findings may be influenced by differences in tissue source, genetic background, medication exposure, disease stage, and analytical methods. Therefore, methylation abnormalities in CACNA1C, CACNB2, KCNQ2, and KCNQ3 should be interpreted as candidate mechanisms for altered neuronal excitability rather than established causal drivers of manic or depressive episodes.

At the circuit level, ion-channel abnormalities may link methylation-associated neurotransmitter dysregulation with altered neuronal excitability in BD. Changes in calcium-channel regulation may affect neurotransmitter release and activity-dependent gene transcription, whereas potassium-channel alterations may influence neuronal repolarization and excitability homeostasis. In this context, ion-channel-related methylation changes may interact with monoaminergic, glutamatergic, and GABAergic abnormalities rather than acting as an independent pathological pathway.

## 6 Circadian rhythm disruption

Circadian rhythm disruption involves the synergistic dysregulation of multiple genes and pathways. Core clock genes, including *PER3*, *CLOCK*, *BMAL1*/*ARNTL*, *NPAS2*, and the melatonin receptor gene *MTNR1A*, together maintain normal circadian rhythms and physiological homeostasis. Disruption of the rhythm not only affects the sleep-wake cycle but also induces various physiological and psychological issues. Abnormalities in sleep-wake patterns are often one of the key clinical features of BD and are crucial for clinical judgment and assessment (Dollish et al., 2024). Salvatore et al. (2008) reported that individuals with BD during manic episodes often exhibit delayed sleep onset, shortened nocturnal sleep, and reduced activity rhythms, indicating circadian rhythm imbalance. This imbalance is considered one of the major mechanisms underlying the typical symptoms of BD, such as sleep disturbances, emotional instability, and other altered physiological processes (Rohr and McCarthy, 2022). A network-wide examination of circadian rhythms shows a more significant connection with BD (McCarthy et al., 2012).

*PER3* is involved in the transcription-translation negative feedback loop of the circadian rhythm. Its transcription is activated by *CLOCK*/*BMAL1*, and the encoded protein interacts with *CLOCK*/*BMAL1* to suppress the expression of core clock genes and downstream clock-controlled genes, thereby maintaining the 24-h cycle (Chen et al., 2024; Li et al., 2024). Among the core clock genes, *CLOCK* is a key transcriptional activator that forms a heterodimer with *BMAL1* (Zeng et al., 2024). Clinically, individuals with BD commonly present with reduced sleep need, insomnia/hypersomnia, changes in appetite, and emotional episodes. Studies in mice have shown that Clock gene mutations can induce lithium-sensitive manic-like behavior, presenting with hyperactivity, reduced sleep, depression-like behaviors, and decreased anxiety-phenotypes resembling aspects of human mania (McCarthy et al., 2012; Roybal et al., 2007).

At the epigenetic level, studies suggest that the methylation level of the *CLOCK* promoter may be elevated in individuals with BD, suppressing its transcription and impairing circadian rhythm function. *BMAL1*/*ARNTL* plays a crucial role in maintaining circadian rhythms by forming a dimer with *CLOCK* to initiate the transcription of PER/CRY. *BMAL1*-deficient mice exhibit rhythmic cycle abnormalities, disrupted sleep patterns, and reduced stability, making it difficult to maintain normal sleep-wake rhythms (Laposky et al., 2005). Bengesser et al. found (Bengesser et al., 2018) that the methylation level of the cg05733463 CpG site in the *BMAL1* gene was significantly higher in individuals with BD, suggesting that the epigenetic alteration at this site may be associated with circadian rhythm disruption or the pathophysiological mechanisms of BD.

Melatonin is secreted by the pineal gland and is regulated by the suprachiasmatic nucleus (SCN), transmitting “time signals” to the body. It is involved in multiple physiological processes, including sleep, appetite, and reproduction, and serves as an important mediator for circadian rhythm and sleep-wake regulation (Chung et al., 2024). *MTNR1A*, a type 1 melatonin receptor, is one of its key effector receptors. Studies have shown (Lesicka et al., 2023) individuals with BD exhibit significantly lower methylation levels of the *MTNR1A* gene compared to healthy controls, suggesting that its epigenetic abnormality may contribute to melatonin signal imbalance and rhythm disturbance.

Moreover, DNA methylation regulating clock genes may also indirectly associate with DA system abnormalities. Under normal conditions, DA activity exhibits circadian rhythmic fluctuations. However, in clock gene mutant mice, abnormal daytime DA activity peaks and behavioral disturbances are observed, which are associated with increased activity and manic-like behavior during the day, suggesting that Clock deficiency may be associated with altered DA activity rhythms, potentially disrupting normal day–night behavioral patterns (Sidor et al., 2015). In individuals with BD, increased *CLOCK* promoter methylation may reduce *CLOCK* expression and affect *CLOCK*-*BMAL1*-mediated circadian gene transcription, thereby potentially altering dopaminergic neuronal rhythmicity.

Overall, circadian rhythm disruption in BD may involve epigenetic alterations across multiple clock- and melatonin-related genes. CLOCK promoter hypermethylation may reduce CLOCK expression and weaken the CLOCK–BMAL1 transcriptional complex, thereby affecting downstream circadian regulators such as PER3. Increased methylation at specific BMAL1/ARNTL sites and decreased methylation of MTNR1A may also be associated with altered circadian and melatonin signaling. These changes may contribute to sleep–wake instability, delayed sleep onset, reduced sleep duration, and mood-state fluctuations in BD. Importantly, circadian methylation changes may interact with dopaminergic rhythmicity rather than acting as an isolated pathway. However, direct evidence linking specific methylation changes in clock genes to clinical phase transitions remains limited and requires longitudinal validation.

Circadian disruption may also interact with immune-inflammatory and neurotransmitter pathways in BD. Sleep-wake instability and clock-gene dysregulation may influence stress-response systems, dopaminergic rhythmicity, and inflammatory signaling. Conversely, inflammatory cytokines may affect synaptic function, neuroplasticity, and epigenetic regulation. This bidirectional interaction suggests that circadian and immune-inflammatory abnormalities may jointly amplify mood-state instability through shared effects on methylation regulation, neurotransmission, and neural circuit plasticity.

## 7 Immune inflammation

Immune-inflammatory dysregulation may represent an important component of BD pathophysiology. Tumor necrosis factor-alpha (TNF-α), a key pro-inflammatory mediator, is significantly elevated in BD and other psychiatric disorders (Kauer-Sant’Anna et al., 2009). TNF-α can activate downstream signaling pathways through TNFR1/TNFR2 and may be involved in apoptosis-related and inflammatory responses. Its prolonged elevation may be associated with impaired neuroplasticity and cognitive dysfunction (Brietzke and Kapczinski, 2008; Millett et al., 2020). Misiak et al. (2020) further demonstrated that TNF-α, IL-6, and soluble receptors sTNF-R1 and sIL-2R are significantly elevated in the serum of individuals with BD, with even higher levels observed during depressive and manic episodes.

It is important to note that the body has an endogenous negative feedback mechanism to limit excessive inflammation, where the cytokine signaling suppressor (SOCS) family plays a role by inhibiting the JAK/STAT pathway and mediating immune regulation. (Aytac et al., 2023; Keshavarzi et al., 2019) reported that the methylation level of the SOCS1 gene promoter is higher in individuals with BD compared to healthy controls, and is closely related to the −1478 CA/del polymorphism. Moreover, SOCS1 mRNA expression is significantly negatively correlated with its methylation level, suggesting that epigenetic silencing weakens SOCS1’s anti-inflammatory function. In this context, long non-coding RNA HCG9 is considered to be involved in immune regulation and immune response. Studies found (Pal et al., 2016) that HCG9 exhibits significant and consistent hypomethylation in individuals with BD, a pattern observed not only in the prefrontal cortex, which is associated with mood and cognition, but also in peripheral blood samples. Low methylation of HCG9 may be associated with its upregulation and with increased T Cell and monocyte/macrophage activation, as well as the production of pro-inflammatory cytokines such as IL-6, TNF-α, and IFN-γ. These changes, under conditions of SOCS1 dysfunction, may contribute to immune-inflammatory dysregulation in BD.

In summary, immune-inflammatory abnormalities in BD may involve a shift toward pro-inflammatory activation with impaired negative regulation, although the underlying epigenetic evidence remains largely associative. Elevated TNF-α, IL-6, sTNF-R1, and sIL-2R may be associated with altered neuroplasticity and mood-state activity, whereas SOCS1 promoter hypermethylation may weaken negative regulation of the JAK/STAT pathway. HCG9 hypomethylation may also be related to pro-inflammatory immune activation. Rather than representing a separate immune module, inflammation-related methylation changes may interact with neurotrophic, glutamatergic, circadian, and stress-response pathways. Therefore, SOCS1- and HCG9-related methylation abnormalities should be interpreted as candidate contributors to immune dysregulation in BD rather than definitive causal markers of chronic inflammation.

## 8 Candidate DNA methylation signatures for BD/MDD differentiation and disease-burden research

Because DNA methylation findings in BD come from different sample sources, their translational meaning should be interpreted with caution. Findings from postmortem brain tissue mainly provide mechanistic information about disease-relevant brain regions, whereas methylation changes detected in peripheral blood, peripheral blood mononuclear cells, or patient-derived cellular models may be more relevant to future clinically accessible biomarker research. Therefore, the methylation patterns discussed in this section are considered candidate signatures for research rather than established diagnostic, prognostic, or treatment-guiding biomarkers.

### 8.1 Candidate methylation signatures for BD/MDD differentiation

BD and Major Depressive Disorder (MDD) exhibit overlapping clinical manifestations during the depressive phase, including low mood and loss of interest. Therefore, accurate differentiation is crucial for diagnosis and treatment. A key distinction between the two disorders lies in BD’s characteristic mood swings, including mania, hypomania, and depression, representing emotional “poles.”

At the epigenetic level, there are distinguishable differences in the DNA methylation profiles of BD and MDD. Early studies revealed that BD’s methylation patterns are more complex, fluctuating significantly with mood phases (mania/depression), whereas MDD is typically characterized by a more stable, higher methylation level. For example, in BD, the *BDNF* and *SLC6A4* promoters show lower methylation during mania and higher methylation during depression, while MDD exhibits a consistently high methylation level (Fries et al., 2016). Further research indicated (Ludwig and Dwivedi, 2016) that during the depressive phase, both BD and MDD show an increase in *BDNF* methylation, but the phase dependency is more pronounced in BD, whereas MDD primarily exhibits consistently high methylation. Li M et al. (2019) supported these findings and suggested that methylation differences related to depression show consistency, with BD exhibiting a “patchwork” pattern of both low and high methylation at specific sites across the genome, whereas MDD tends to show globally high methylation. Bundo et al. (2021) found that in the prefrontal cortex (PFC), individuals with BD exhibited global low methylation in neurons and astrocytes, but certain genes, such as *NTRK2* and *GRIN1*, showed high methylation in neurons, suggesting a “global low—local high” fine-grained feature of BD. Beyond methylation, recent metabolomics studies also revealed differences between the two disorders. BD’s metabolic profile fluctuates dramatically with mood phases (e.g., increased energy metabolism products during high-energy states), whereas MDD’s metabolic profile remains more stable (Tomasik et al., 2024). From a genetic heterogeneity perspective, BD consists of multiple subtypes with significant genetic differences, with biomarkers specific to mania that are not shared by MDD (Charney et al., 2017).

In summary, BD and MDD show overlapping depressive symptoms but may differ in mood-state dynamics and disease-associated biological patterns. Current evidence suggests that BD may present more dynamic and phase-dependent methylation changes, whereas MDD has been associated with relatively more stable methylation alterations in some studies. Methylation patterns involving BDNF, SLC6A4, NTRK2, GRIN1, and genome-wide profiles may therefore provide candidate molecular signatures for studying BD/MDD differentiation. However, these signatures should not be interpreted as clinically validated diagnostic criteria, because reported findings may be influenced by tissue source, mood state, medication exposure, cohort composition, and analytical methods. Thus, DNA methylation differences are better understood as exploratory tools for disease stratification research rather than stand-alone biomarkers for clinical diagnosis.

### 8.2 Candidate DNA methylation-based indicators for disease burden and prognosis-related research

Individuals with BD not only experience long-term psychiatric symptoms but also face increased physical health risks and reduced life expectancy. Their overall mortality risk is approximately 2.02 times higher than that of the general population, with a reduced life expectancy of approximately 13 years and a suicide mortality risk approximately 11.69 times higher (Bourdon et al., 2023; Biazus et al., 2023; Chan et al., 2022). These adverse outcomes have prompted interest in biological aging markers such as epigenetic age acceleration (EAA), although the directionality and clinical significance of this relationship remain unclear. Bourdon et al. (2023) reported that individuals with BD may show evidence of EAA, with different epigenetic clocks showing varying sensitivity to aging-related changes. Individuals with more frequent and severe mood episodes appear to show more pronounced EAA, whereas long-term use of mood stabilizers may attenuate this acceleration (Okazaki et al., 2020). Repeated mood episodes have also been associated with increased mitochondrial DNA copy number and mitochondrial dysfunction, which may contribute to cellular senescence (Fries et al., 2017). Jeremian et al. (2022) reported that individuals with multiple depressive or manic episodes showed greater EAA, suggesting that EAA may represent a candidate indicator of illness burden or biological aging in BD. Fries et al. (2026) further reported that individuals with BD and EAA had a reduced cerebrospinal fluid (CSF) Aβ42/40 ratio, suggesting a possible association with early neurodegenerative pathological changes. A lower Aβ42/40 ratio may reflect increased β-amyloid deposition in the brain, and its association with EAA may indicate a potential link between epigenetic aging and cognitive decline risk.

Therefore, EAA may be considered a candidate DNA methylation-based indicator for studying illness burden, biological aging, and neurodegenerative risk in BD. However, whether EAA represents a cause, consequence, or correlate of disease progression remains unclear. Long-term mood stabilizer exposure, inflammatory status, metabolic changes, lifestyle factors, and repeated mood episodes may all influence epigenetic aging estimates. Thus, although EAA has potential value for disease-stage characterization and prognosis-related research, its application in treatment monitoring or clinical decision-making requires further longitudinal validation.

## 9 DNA methylation and treatment-response research

In individuals with BD, mood-state changes and long-term pharmacological exposure may both influence DNA methylation patterns. Asai et al. (2013) reported that treatment with mood stabilizers was associated with reduced methylation at selected CpG sites in human neuroblastoma SK-N-SH cells with high SLC6A4 methylation, suggesting that mood stabilizers may modulate methylation patterns related to serotonergic regulation. Backlund et al. (2015) found that peripheral blood DNA methylation levels decreased during lithium monotherapy but increased during combined lithium and valproate treatment, indicating that different pharmacological regimens may have distinct effects on peripheral methylation profiles. Antipsychotic drugs have also been investigated in relation to epigenetic regulation. Houtepen et al. (2016) reported that olanzapine, clozapine, and quetiapine may induce demethylation at selected gene promoters, including BDNF and SLC6A4, which could influence gene-expression pathways related to neuroplasticity and neurotransmitter regulation.

However, the clinical significance of these drug-associated methylation changes remains uncertain. Existing evidence is limited by *in vitro* models, peripheral tissue measurements, medication-history confounding, disease-state effects, and lack of longitudinal validation. Therefore, methylation changes associated with mood stabilizers or antipsychotics should be interpreted as potential mechanisms related to treatment response rather than direct evidence that these drugs improve BD symptoms through methylation remodeling.

Although DNA methylation inhibitors can theoretically modify abnormal methylation states, their application in BD remains highly experimental. Major challenges include target specificity, off-target effects, cell-type and brain-region selectivity, blood–brain barrier delivery, dose control, long-term safety, and the risk of disrupting physiological methylation programs. Therefore, methylation-targeted interventions should be discussed as a future research direction rather than an established precision treatment strategy for BD.

## 10 Limitations and future directions

Several limitations should be considered when interpreting current evidence on DNA methylation in BD. First, most available studies are cross-sectional and associative, making it difficult to determine whether methylation changes precede disease onset, reflect mood-state transitions, or arise as consequences of illness progression, medication exposure, or environmental factors. Second, many studies rely on peripheral blood or mixed-cell samples, which may not fully reflect methylation regulation in disease-relevant brain regions or specific neuronal and glial cell populations. Differences in neuron–glia composition, immune-cell proportions, and peripheral blood cell-type composition may substantially influence methylation estimates. Third, sample sizes are often limited, and study populations differ in age, sex, genetic background, smoking status, metabolic status, disease stage, mood state, medication exposure, comorbidity, and environmental stressors. These factors may confound observed methylation differences and limit cross-study reproducibility.

Methodological heterogeneity also remains a major challenge. Differences in methylation detection platforms, CpG-site coverage, bisulfite-conversion efficiency, sequencing depth, batch effects, normalization procedures, statistical models, and correction for multiple testing may contribute to inconsistent findings across studies. In addition, cell-type deconvolution methods can only partially correct for tissue heterogeneity, particularly when disease-relevant cell populations are underrepresented or when reference methylomes are incomplete. These technical issues are especially important in psychiatric epigenetics, where disease-relevant tissues are difficult to obtain and peripheral methylation findings may not directly reflect brain-region-specific regulation.

Future research should prioritize longitudinal designs, brain-region-specific and cell-type-resolved methylome analyses, and integration with transcriptomic, proteomic, metabolomic, neuroimaging, and clinical data. Single-cell and spatial epigenomic approaches may help clarify whether methylation alterations occur in specific neuronal or glial populations and how they relate to circuit-level dysfunction. In addition, future studies should distinguish mood-state-dependent methylation changes from trait-like epigenetic signatures and treatment-related effects. Large, independent, multi-center cohorts with standardized protocols are needed to evaluate the reproducibility, tissue specificity, and translational relevance of candidate methylation markers. Such studies will be essential for determining whether DNA methylation changes are causal contributors, compensatory responses, or secondary correlates of BD pathophysiology.

## 11 Conclusion

BD is a complex and clinically heterogeneous psychiatric disorder involving interacting abnormalities in neurotransmitter systems, neuroplasticity, ion-channel excitability, circadian rhythm, and immune-inflammatory regulation. Current evidence suggests that DNA methylation may represent a dynamic epigenetic regulatory layer through which genetic vulnerability, environmental exposure, mood-state changes, and pharmacological treatment influence these biological systems. Methylation alterations in genes related to dopamine, serotonin, glutamate, GABA, BDNF/TrkB signaling, ion channels, clock regulation, and immune-inflammatory pathways may provide biologically plausible mechanisms for BD-related neural circuit instability.

However, most available evidence remains associative rather than causal. DNA methylation changes should therefore be interpreted as candidate mechanisms and exploratory translational signatures rather than established diagnostic markers or therapeutic targets. Candidate methylation profiles, including BDNF-related methylation changes, GRIN2B CpG-site alterations, and EAA, may have potential relevance for disease stratification and treatment-response research, but their clinical utility requires further validation. Similarly, methylation-targeted interventions remain experimental and face substantial challenges related to specificity, safety, delivery, and clinical applicability. Future longitudinal, brain-region-specific, cell-type-resolved, and multi-omics studies are needed to clarify the mechanistic and translational significance of DNA methylation in BD.
